# Time Sensitivity Factor of Single Pulmonary Nodule: A New Cancer Characteristic Metabolic Parameter by ^18^ **F**-FDG PET

**DOI:** 10.1155/2014/830135

**Published:** 2014-05-18

**Authors:** Ching-Yuan Cheng, Kwo-Whei Lee, Chiang-Hsuan Lee, Yeu-Sheng Tyan, Cheng-Yi Cheng, Jhi-Joung Wang, Chao-Wei Yang, Wen-Sheng Huang, Ching-Yee Oliver Wong

**Affiliations:** ^1^Department of Chest Surgery, Changhua Christian Hospital, Changhua, Taiwan; ^2^Department of Radiology, Changhua Christian Hospital, Changhua, Taiwan; ^3^Departments of Nuclear Medicine and Anesthesiology, Chi-Mei General Hospital, Tainan, Taiwan; ^4^Department of Radiology, Chung Shan Medical University Hospital, Taichung, Taiwan; ^5^Department of Nuclear Medicine, Tri-Service General Hospital, Taipei, Taiwan; ^6^Department of Nuclear Medicine, Changhua Christian Hospital, 135 Nan-Hsiao Street, Changhua 50006, Taiwan; ^7^Department of Nuclear Medicine, Oakland University William Beaumont Hospital, 3601 W. Thirteen Mile Road, Royal Oak, MI 48073-6769, USA

## Abstract

*Objective*. To calculate the time sensitivity factor (*S*) for discriminating the solitary pulmonary nodule (SPN) by FDG PET at different time points. *Methods*. The multiple time-point FDG PET images from 41 patients for evaluating SPN seen on chest X-ray or CT were prospectively analyzed to calculate and evaluate *S* against the gold standard of tissue histology (*n* = 38) or long term clinicoradiographic follow-up (*n* = 3). The maximal standardized uptake values (SUV) at the 3 hourly time points were measured. The *S* was calculated using *S* = *d*{ln⁡(SUV)}/*d*{ln⁡(*t*)} at 3 different time intervals. ROC analysis of the *S* parameters was performed to evaluate the optimal cut-off value and their accuracy in classifying the SPN. *Results*. The SUV in malignant SPN was higher than the corresponding value in benign lesions at all 3 hourly time points (*P* < 0.003). The *S* parameters using 3 different time intervals all significantly separated the two groups (*P* < 0.0005) with an optimal cut-off point near the theoretical value of zero with a high sensitivity of 100% and specificity of 86%. *Conclusion*. The *S* can be calculated for SPNs using multiple time-point FDG PET, providing a tumor characteristic metabolic parameter with high discrimination power using a simple positive value representing malignancy.

## 1. Introduction

A solitary pulmonary nodule (SPN) is defined as an intraparenchymal lung lesion that is less than 3 cm in diameter and without associated atelectasis or adenopathy [1]. The differential diagnosis of a SPN includes neoplastic, infectious, inflammatory, vascular, traumatic, and congenital lesions [2]. The incidence of malignancy in patients with SPN ranges from 10 to 70 percent [3, 4]. Positron emission tomography (PET) with 2-deoxy-2-[18F]-fluoro-D-glucose (FDG) has proven to be valuable for the assessment of SPN. The reported sensitivity and specificity of FDG PET in differentiating malignant from benign nodules ranged from 83% to 97% and from 69% to 100%, respectively [5]. Although highly sensitive, FDG-PET has only intermediate specificity for malignancy [6–9] using traditional semiquantitative technique of standard uptake value (SUV). False-positive FDG-PET scans are known to occur in infectious granulomatous lesions arising from tuberculosis, histoplasmosis, aspergillosis, and noninfectious granulomas including sarcoidosis [10]. Available data indicated that the uptake of FDG in malignancy showed persistent increase for hours after FDG injection [10–13]. Hamberg et al. observed that the tumor concentration of FDG in patients with lung cancer might not reach a plateau within 90 min of imaging; the projected plateau appeared at about 5 h after injection [11]. It appears that the optimal timing of FDG PET for specific tumor types remains to be explored [14]. Review of the published data on the temporal profile of FDG uptake suggests that it is reasonable to use a power function for approximating the FDG uptake over a limited time range [11]:

(1) $$\begin{array}{c} \text{SUV}=kt^{S} \end{array}$$

for some *k* and *S* values to be determined by the tumor specific data. The *k* values range from 2.9 to 3.7 and *S* ranges from 0.14 to 0.28 when *t* is measured in minutes by regression analysis of the lung cancer data provided in the literature [11, 14]. With this approximation, the time sensitivity factor can also be represented in a logarithmic differential form [14]:

(2) $$\begin{array}{c} S=\frac{d\left\{\ln\left(\text{SUV}\right)\right\}}{d\left\{\ln\left(t\right)\right\}}. \end{array}$$

Here the *S* represents fractional (percentage) change of SUV over the fractional (percentage) change of time and is a dimensionless exponent quantity. It is not simply the slope (where there are time units) of tumor FDG uptake as revealed by expanding the above definition:

(3) S={d(SUV)dt}{tSUV}=slope  of  FDG  uptake×{tSUV}.

For discrete time point data, the *S* value of a particular patient can be obtained by

(4) $$\begin{array}{c} \frac{S_{i}}{S_{j}}=\frac{\ln\left({\text{SUV}}_{i}/{\text{SUV}}_{j}\right)}{\ln\left(t_{i}/t_{j}\right)} \end{array}$$

for any given two time points (*t*_*i*_, *t*_*j*_). Within a given time range for which the data is sampled, the commonly used time dependable retention index (RI) in percentage can be derived from known *S* value by

(5) RIiRIj={SUViSUVj−1}×100%={(titj)S−1}×100%.

Thus, the time sensitivity factor incorporates the retention index and the potential variation in time selection in the delayed FDG imaging as evident by the following equation:

(6) $$\begin{array}{c} \frac{S_{i}}{S_{j}}=\frac{\ln\left(1+\left({\text{RI}}_{i}/{\text{RI}}_{j}\right)\right)}{\left\{\ln\left(t_{i}\right)-\ln\left(t_{j}\right)\right\}}. \end{array}$$

The time sensitivity factor is not simply retention index [12, 13] as it adjusts for variation in time intervals for obtaining the SUVs. The *S* factor is simple tumor kinetic factor which may be another characteristic metabolic parameter related to the tumor metabolism or phenotype (mainly the hexokinase II activity) [14]. Traditionally for FDG-PET imaging, SUV is obtained at some standardized single-scan time, such as one hour after injection, which will be influenced by both the actual uptake time and partial volume effects. A second delayed SUV may be arbitrarily chosen to be half an hour to three hours after injection. The purpose of this study was thus to explore and calculate the time sensitivity factor (*S*) in known malignant and benign single pulmonary nodules for obtaining a tumor characteristic metabolic parameter incorporating the time and size variations by F-18 FDG PET imaging.

## 2. Materials and Methods

Forty-one consecutive patients (23 men, 18 women; mean age 60 years; age range 36–82 years) with SPN seen on chest radiography or CT were prospectively enrolled for this study. All patients underwent FDG-PET within 2 weeks after the detection of SPN. Final diagnoses were made based on histology of biopsy specimens or at least 12 months of clinicoradiographic (CT) follow-up. A lesion that demonstrated no evidence of malignancy within the 12-month follow-up period was classified as benign as in the literature [15]. This study was approved by the institutional review board with informed consents from all patients.

All FDG PET scans were acquired with a dedicated PET system (ECAT Exact HR+ PET camera; Siemens/CTI, Knoxville, TN, USA) using a full-width at half-maximum of 4.5 mm and a transaxial field of view of 15 cm. All patients had fasted at least 6 h with serum glucose levels measured before FDG injection to be less than 150 mg/dL. After intravenous injection of an average of 370 MBq (10 mCi) of F-18 FDG, the patients were kept at rest in a quiet, dimly lit room for 60 min. Three sets of FDG PET images were acquired from patients in a supine position at one hour (scan 1), two hours (scan 2), and three hours (scan 3) after injection using two-dimensional acquisition mode. The emission and transmission scans were obtained in an alternating sequence per bed position. A transmission scan was obtained with all sets of images for attenuation correction with a ^68^Ge source [16, 17]. Reconstruction of both transmission and emission images was performed with an iterative ordered-subsets expectation maximization (OSEM) algorithm with 4 iterations and 8 subsets.

The ^18^F-FDG PET scans were analyzed semiquantitatively by a consensus approach of two experienced nuclear medicine physicians who were unaware of the final diagnosis. For all PET images scanned at 1, 2, and 3 hours, circular regions of interest (ROI) were placed in the central portion of the lesion on the transaxial slices showing their highest activity. For each ROI, the maximal standardized uptake value (SUV), defined as the highest measured activity within the ROI per injected activity per unit body mass, was determined. For each SPN, six parameters: SUV_1 h_, SUV_2 h_, SUV_3 h_, *S*_2 h/1 h_, *S*_3 h/1 h_, *S*_3 h/2 h_ at 3 time points and time intervals, respectively, were calculated for statistical analyses.

Boxplot exploration of the calculated *S* parameters and subsequent ROC analysis were performed using SPSS for Windows (SPSS, Inc., Chicago, IL). A *P* value of less than 0.05 was considered significant.

## 3. Results

**Figure 1:**
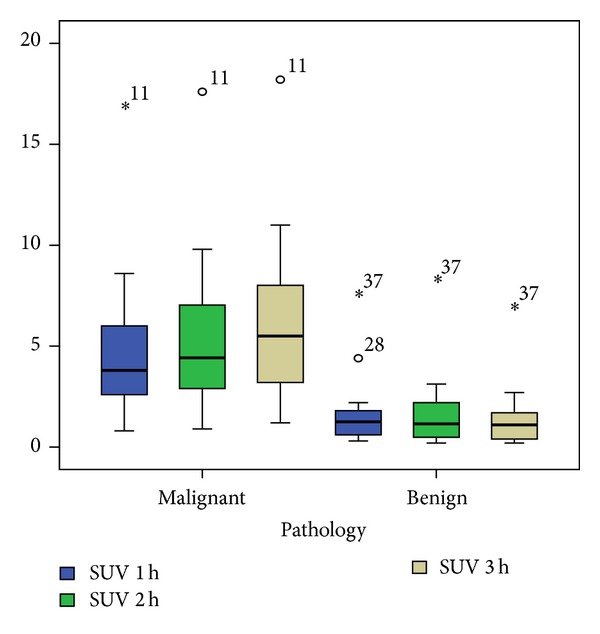
Boxplots of SUV at various time points at 1, 2, and 3 hours. Note the outliners as indicated by the case numbers.

**Figure 2:**
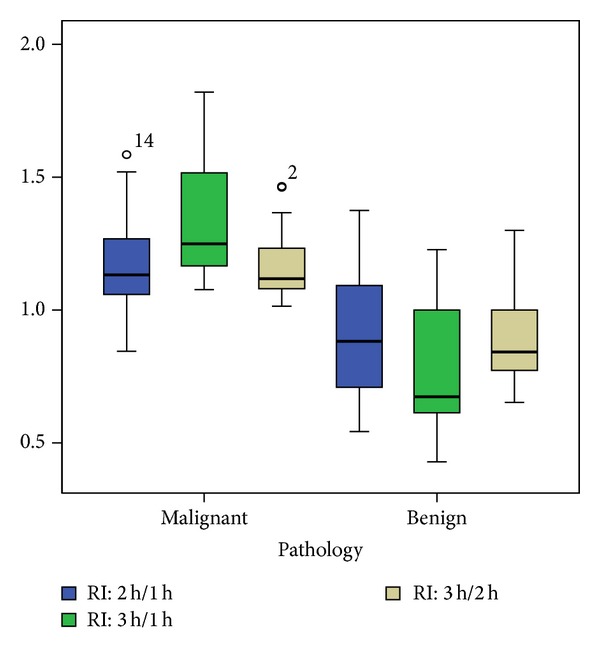
The boxplots of the retention index (RI) at various time intervals from scans at 1, 2, and 3 hours after injection. Note the outliners as indicated by the case numbers.

**Figure 3:**
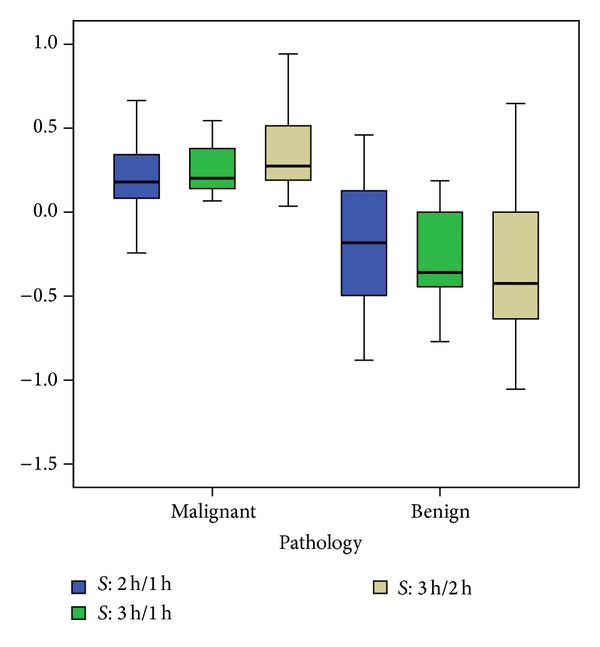
The boxplot of the time sensitivity factors in discriminating the malignant and benign SPNs. Note the separation between these two groups without outliners as compared to SUV and RI in Figures 1 and 2, respectively.

**Figure 4:**
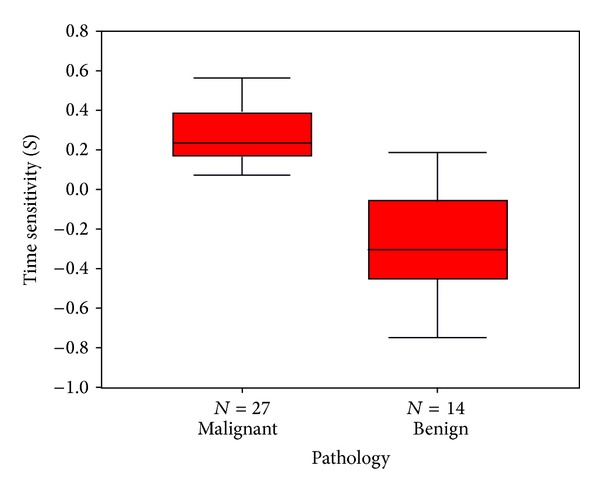
The boxplot of the average time sensitivity factor (*S*) in discriminating the malignant and benign SPNs. Note the clear-cut wide separation between these two groups at around zero value of *S* without any outliners as compared to SUV and RI in Figures 1 and 2, respectively.

**Figure 5:**
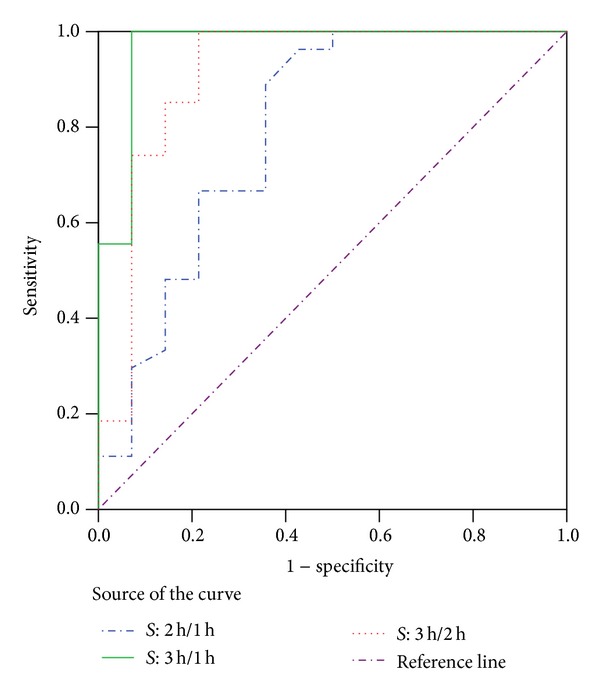
The ROC analysis of time sensitivity factors in discriminating malignant and benign SPNs.

Among the 27 cancerous SPNs, there were 16 adenocarcinomas, 3 bronchioloalveolar carcinomas (BAC), 7 squamous cell carcinomas, and 1 undifferentiated large cell carcinoma. The pathology for the 14 benign SPNs was inflammation (5), organizing pneumonia (4), granuloma (1), sclerosing hemangioma (1), and clinicoradiological stable lesions (3). The average SUV in malignant SPN (mean ± SD) was 4.6 ± 3.3 at 1 hr, 5.3 ± 3.5 at 2 hr, and 6.0 ± 3.7 at 3 hr, which are all significantly higher than the corresponding values in benign lesions (1.8 ± 2.0 on 1 hr scan, 1.7 ± 2.1 on 2 hr scan, and 1.5 ± 1.8 on 3 hr scan with *P* = 0.002, <0.0005, <0.0005, resp.) (Figure 1). Some outliners were noted as indicated by case numbers in Figure 1. Similarly, the RI in malignant SPNs (mean ± SD) was higher than the corresponding values in benign lesions (1.17 ± 0.16 versus 0.91 ± 0.24, *P* = 0.002 on scans at 1 and 2 hr, 1.37 ± 0.23 versus 0.79 ± 0.23, *P* < 0.0 005 on scans at 1 and 3 hr, and 1.16 ± 0.13 versus 0.88 ± 0.18, *P* < 0.0005 on scans at 2 and 3 hr) (Figure 2). Again a few outliners were noted in Figure 2. The time sensitivity factors, *S*_2 h/1 h_,   *S*_3 h/1 h_,   *S*_3 h/2 h  _, calculated using various time intervals (1 to 2 hours) using scans 1, 2, and 3, were also significantly different between malignant (positive mean) and benign (negative mean) lesions (0.21 ± 0.20 versus −0.18 ± 0.39 on scans at 1 and 2 hr, 0.27 ± 0.15 versus −0.25 ± 0.27 on scans at 1 and 3 hr, and 0.36 ± 0.26 versus −0.37 ± 0.47 on scans at 2 and 3 hr with *P* < 0.0005 for all 3 time intervals) (Figure 3). No outliners were noted (Figure 3) for all the three time intervals used. There was a clear reversal in sign between malignant (average *S* = +0.28 ± 0.26) and benign (average *S* = −0.27 ± 0.26) lesions (*P* < 0.0005) and the separation between these two groups (Figure 4) was clearly better compared to SUV and RI (Figures 1 and 2). The ROC analysis showed similar areas under curves (AUC) of *S*_3 h/1 h_ (AUC = 0.968), *S*_3 h/2 h  _ (AUC = 0.913), but better than *S*_2 h/1 h_ (AUC = 0.795) (Figure 5), suggesting the relative importance of the 3 hr delayed scan data. The sensitivity and specificity of the averaged *S* for malignancy were 100% and 86%, respectively, and the optimal cut-off value for the averaged *S* was 0.06, which was very close to the theoretical value of zero.

## 4. Discussion

The preliminary data suggested that the time sensitivity factor for non-small lung cancers could be calculated to show a distinct positive value compared to the negative value in benign lesions by using multiple delayed PET scans for measurement of SUV. The maximal SUV (SUV_3 h_) at 3 hours provided the most valuable information in time sensitivity factor calculation in discriminating benign from malignant lesions as revealed by *S*_3 h/1 h_ which had the highest accuracy value by ROC analysis. Thus, the calculated time sensitivity factor requires scans from a much later time point than the traditionally used 1- and 2-hour delayed scans. The theory behind SUV as a semiquantitative value was reviewed by Huang et al. [17] and has been found to be of great significance in determining pulmonary lesions [18, 19]. Currently the SUV value (SUV_1 h_) at 1 hour image of 2.5 was usually used as a cut-off point in differentiating malignant from benign pulmonary lesions and it had produced good accuracy [6, 7, 9, 20, 21]. However, a single SUV value will be influenced by partial volume effects and many other unknown factors, causing false negative and false positive results as seen in the results of many prior studies [6, 7, 9, 20, 21]. Some slow-growing malignancies such as BAC and carcinoid tumors may have a lower avidity for F-18 FDG while infectious lesions such as tuberculosis, commonly found in Asia, inflammation, and granulomas can have a high F-18 FDG uptake mimicking pulmonary malignancy [10, 12, 22]. In current study, the accuracy of SUV_1 h_ was only 68%, much lower than any *S* parameters evaluated.

Other previous reports suggested that delayed image at 2 hours is helpful in detecting pulmonary malignancy based on the theory that malignant pulmonary is more capable of harvesting F-18 FDG than benign pulmonary nodules [10]. Therefore, if an uprising SUV at 2-hour image is measured to compare with that from 1-hour image, a more F-18 FDG avidity is revealed, which may suggest higher likelihood of malignant pulmonary nodules. Kernstine et al. reported a sensitivity of 100% and specificity of 89% when adopting a 10% increase in SUV as a possible cut-off value [23]. The current study demonstrated a tendency of increasing SUV in malignant pulmonary nodule similar to prior studies by Demura et al. [12]. However, the current study has its unique nature by summarizing all variables to be reflected in the positive value of *S*. For instance, the calculated RIs based on the *S* determination for various time points such as 0.5–1 hr, 1-2 hr, 1.5–2 hr, 1–3 hr, and 2-3 hr scan for malignancy would be 21%, 21%, 8%, 36%, and 12%, respectively. These values, though theoretical, reveal markedly different values if various different time points are used clinically. Thus, if half-hour delayed FDG scans are obtained during evaluation of head and neck tumors [14], the clinicians have to deal with a different number when 1-hour delayed FDG scans are more feasible and commonly used in SPN.

To fully investigate the behavior of malignant pulmonary nodule, the current study measured the SUV max of the pulmonary nodules up to 3 hours after injection and the time sensitivity factors were calculated to incorporate different time intervals used. The optimal cut-off value of the average *S* value was 0.06 (almost identical to the theoretical value of zero) and produced the best separation by boxplot analysis with 100% sensitivity and 86% specificity. The *S*_3 h/1 h_ and *S*_3 h/2 h_ achieved higher accuracy than *S*_2 h/1 h_, suggesting the usefulness of later time-point scans for calculating the *S* values for a given patient using multiple time points [24].

Benign lesions in current study tended to have a lower and negative *S* value than malignant lesions because the former had a trend of decreasing SUV max with time. Only two benign lesions had *S* value slightly higher than zero (i.e., positive value). It thus appeared that *S* value from delayed images up to 3 hours provided valuable information in determining malignancy of SPNs as there were no false negative cases in the current study.

Malignant nodules tended to have a higher sensitivity to changes in time while benign nodules showed the reverse trend. By adopting a simple positive *S* value, all malignant lesions were detected in the current study. It thus suggested that the newly defined *S* value might be helpful in discriminating the nature of SPNs in patients with equivocal PET results by the standard single 1-hour scan.

Taking advantage of measuring the kinetics of glucose metabolic rate in pulmonary nodules by using a simple parameter incorporating the time sensitivity factor like the *S* value, PET may be better recognized as an accurate modality in the evaluation of pulmonary malignancy or its tumor metabolic phenotype. This is largely because of its elimination of partial volume effects (size variation) and time factors (imaging interval variations). Moreover, the role of PET in determining pulmonary malignancy smaller than 1 centimeter is still unsettled [25]. Yankelevitz and Henschke reported that the latent time for some slow-growing pulmonary malignancies could be more than 2 years [25]. This observation might be especially true for subcentimeter nodules, which produced difficulty in the measurement of volume and density changes by radiological follow-up. Subsequently, slow-growing malignancies could be misclassified as benign lesions. Thus a parameter, like *S*, that is mostly immune to partial volume effects and circumvent SUV_1 h_ less than 2.5, may be helpful for evaluating subcentimeter lesions.

Even though there might have been some modifications in SUV calculation including glucose-corrected SUV, lean body mass correction, and body surface area correction [26–28], the logarithmic ratio used in the current definition of *S* nullified these various controversies in SUV calculations. If one should make a final interpretation of the study solely on a single SUV, rigorous attention to standardize technique is of paramount importance for the evaluation of pulmonary nodules [10, 29, 30]. The SUV is also dependent on the reproducibility of many potential known and unknown factors among scans pertinent to a particular machine or imaging center [17]. Thus, the time sensitivity factor, *S*, explored in the current study with the robust logarithmic ratio definition of *S*, yields a simple cancer characteristic metabolic parameter that may further improve the accuracy of FDG PET in evaluating SPN.

## 5. Conclusion

The time sensitivity factor, *S*, from multiple time-point FDG PET for SPN, can be calculated. By using a simple zero as the cut-off point, it discriminates benign (negative) and malignant (positive) lesions with a high accuracy.
